# Amikacin Population Pharmacokinetics in Critically Ill Kuwaiti Patients

**DOI:** 10.1155/2013/202818

**Published:** 2013-01-30

**Authors:** Kamal M. Matar, Yousef Al-lanqawi, Kefaya Abdul-Malek, Roger Jelliffe

**Affiliations:** ^1^Department of Pharmacology & Therapeutics, Faculty of Pharmacy, Kuwait University, P.O. Box 24923, Kuwait City 13110, Kuwait; ^2^Department of Pharmacy, Al-Amiri Hospital, Ministry of Health, P.O. Box 491, Kuwait City 32005, Kuwait; ^3^Intensive Care Unit, Al-Amiri Hospital, Ministry of Health, P.O. Box 491, Kuwait City 32005, Kuwait; ^4^Laboratory of Applied Pharmacokinetics, School of Medicine, University of Southern California, Los Angeles, CA 90033, USA

## Abstract

Amikacin pharmacokinetic data in Kuwaiti (Arab) intensive care unit (ICU) patients are lacking. Fairly sparse serum amikacin peak and trough concentrations data were obtained from adult Kuwaiti ICU patients. The data were analysed using a nonparametric adaptive grid (NPAG) maximum likelihood algorithm. The estimations of the developed model were assessed using mean error (ME) as a measure of bias and mean squared error (MSE) as a measure of precision. A total of 331 serum amikacin concentrations were obtained from 56 patients. The mean (±SD) model parameter values found were *V*_*c*_ = 0.2302 ± 0.0866 L/kg, *k*_slope_ = 0.004045 ± 0.00705 min per unit of creatinine clearance, *k*_12_ = 2.2121 ± 5.506 h^−1^, and *k*_21_ = 1.431 ± 2.796 h^−1^. The serum concentration data were estimated with little bias (ME = −0.88) and good precision (MSE = 13.08). The present study suggests that amikacin pharmacokinetics in adult Kuwaiti ICU patients are generally rather similar to those found in other patients. This population model would provide useful guidance in developing initial amikacin dosage regimens for such patients, especially using multiple model (MM) dosage design, followed by appropriate Bayesian adaptive control, to optimize amikacin dosage regimens for each individual patient.

## 1. Introduction

Amikacin is an aminoglycoside antibiotic that is most effective against Gram-negative bacteria. Its optimal dosing is highly variable and depends on the site and severity of infection, the susceptibility of the organism, and the body weight and renal function of the patient. 

Like other aminoglycosides, amikacin has a very narrow therapeutic index, and the concentrations needed for optimal efficacy are close to those having a risk of toxicity. Amikacin is a concentration-dependent drug, with the rate of killing of microorganisms being proportional to the drug concentrations achieved in serum, especially peak concentrations [1]. However, abnormally high plasma or serum trough concentrations soon after commencing therapy is often associated with toxicity [1]. 

With multiple daily dosing of amikacin, the specific desired peak concentration for life-threatening Gram-negative sepsis usually ranges between 30 and 50 *μ*g/mL, while the corresponding specific desired trough amikacin concentration ranges between 5 and 10 *μ*g/mL.

Although aminoglycosides are extensively used, the accurate determination of their optimal dosage is complicated by marked intra-and interindividual variability in its pharmacokinetic behavior in patients with normal or abnormal renal function [2]. Management of intensive care unit (ICU) patients often requires general support of failing organs including specific treatment and administration of multiple drugs. All these situations may alter the pharmacokinetics of many drugs such as amikacin in critically ill patients. Large interindividual variations in the pharmacokinetics of amikacin in ICU patients have been described [3, 4]. Thus, amikacin is a drug for which therapeutic drug monitoring (TDM) has a well-established role. Individualized optimal dosage regimens should be designed and implemented as early as possible in therapy to yield maximal efficacy. It has been reported that severe sepsis modifies amikacin kinetics by increasing its apparent volume of distribution of the central compartment and decreasing its elimination rate, probably as a consequence of leaky capillaries and organ failure [5–7].

Population pharmacokinetic techniques are widely used to characterize the interindividual variability of pharmacokinetic parameter values among patients. The objective of the present study was to analyze a representative population of critically ill Kuwaiti patients receiving amikacin therapy using the nonparametric adaptive grid (NPAG) program, in the MM-USCPACK collection [8, 9]. This software has been incorporated into the Pmetrics software, which is now embedded in R.

## 2. Materials and Methods

### 2.1. Study Approval

All data for this study were obtained retrospectively during routine clinical care of ICU Kuwaiti patients. The study was approved by both Health Sciences Center (HSC) Ethics Committee (HSC, Kuwait University, Kuwait) and the Ministry of Health Ethics Committee (Ministry of Health, Kuwait). Informed consent was not needed since the blood samples were collected for routine care and therapeutic drug monitoring (TDM) of their amikacin therapy.

### 2.2. Patient Characteristics

From December 2008 to October 2010, fifty six Kuwaiti patients received amikacin therapy in the ICU of Al-Amiri Hospital in Kuwait and were included in this study. No additional blood samples were taken other than those requested for routine TDM of amikacin. The most frequent clinical conditions in these patients were septicemia (23 patients), pneumonia (6 patients), and severe trauma (27 patients). The patients' demographic data and characteristics are presented in Table 1.

### 2.3. Drug Administration

All patients received an initial standard Kuwaiti amikacin dosage regimen of 500 mg every 12 h. All doses were administered intravenously over 5 min, the standard practice in Kuwait. Exact dosing times were recorded.

### 2.4. Blood Sampling

Blood samples were withdrawn daily from the patients for determination of amikacin in serum. The trough level was taken within 30 min before a dose was administered, whereas the peak level was taken 1 h after the 5 min i.v. infusion was started. Exact blood sampling times were recorded. Blood samples were immediately centrifuged, and serum samples were collected and stored at −80°C pending analysis.

### 2.5. Drug Analysis

Serum amikacin samples as well as serum creatinine were measured by Kobas Integra 400 (Roche Diagnostics, Basel, Switzerland). Calibration standards of amikacin in serum were at concentrations of 0, 2.5, 5, 10, 20, and 40 *μ*g/mL. Quality control samples at concentrations of 5.31, 14.8, and 26.9 *μ*g/mL were assayed each time patient samples were assayed. The tests were performed according to the manufacturer's protocol [11]. The intra-run and interrun coefficients of variation for amikacin assay were less than 5% and 10%, respectively. 

### 2.6. Assay Error

The standard deviation (SD) of the assay over its working range was determined using 5 replicates of each serum amikacin concentrations of 0, 2.5, 5, 10, 20, and 40 *μ*g/mL. The relationship between serum amikacin concentrations and the assay SD was described by a polynomial equation using the MM-USCPACK software as follows:

(1) $$\begin{array}{c} \text{SD}=A_{0}C^{0}+A_{1}C^{1}+A_{2}C^{2}, \end{array}$$

where *A*_0_, *A*_1_, *A*_2_, are various coefficients and *C* ^0^, *C* ^1^, *C* ^2^ are the concentrations raised to the zero power, first power, and the second power, respectively. The coefficients of this polynomial equation were used to give proper weighting of each measured concentration by the reciprocal of the assay variance (SD^2^) at each measured concentration, in performing the population analysis using the NPAG software.

### 2.7. Data Analysis

In the present study, the NPAG method of population pharmacokinetic analysis was selected for performing the population pharmacokinetic modeling for the following specific reasons. In contrast to parametric approaches such as NONMEM, for example, there is no need to make any constraining assumptions about the shape of the model parameter distributions such as normal and log normal. In addition, nonparametric (NP) models permit multiple model (MM) dosage design, which is always maximally precise [12], while dosage regimens developed using only single-point parameter values such as means or medians from parametric population modeling methods cannot do this, as they do not use the entire model parameter distributions and therefore cannot evaluate and maximize the expected precision with which the dosage regimen hits a clinically selected target goal. Moreover, NPAG software calculates the likelihood function exactly and thus possesses statistical consistency in contrast to parametric approaches, many of which (but certainly not all) use only approximate methods; first order (FO), first-order conditional estimation (FOCE), for example, to calculate the likelihood. They, therefore, do not have statistical consistency. This most desirable property of statistical consistency means that the more subjects one studies in the population, the closer the estimated parameter distributions approach the true ones. This means that the more subjects studied, the closer the predicted parameter value approaches the true value [9, 12].

Individual patient data including serum amikacin concentrations, age, weight, height, gender, serum creatinine, and dosage history were entered in the MM-USCPACK PC program. The software used the entire dosing history, amikacin peak and trough levels, and the estimated creatinine clearances [8]. The model pharmacokinetic parameter distributions for the population were computed by the NPAG nonparametric maximum likelihood method [9]. The model used was a two-compartment open model with elimination from the central compartment. The fitted parameters were the apparent volume of distribution of the central compartment (*V*_*c*_, L/kg), the transfer rate constants (*k*_12_ and *k*_21_) from the central to peripheral and from the peripheral back to central compartments, respectively, (in h^−1^), *k*_slope_, the renal component of *K*_el_, the elimination rate constant, (in h^−1^ per unit of creatinine clearance), where

(2) $$\begin{array}{c} K_{\text{el}}=k_{\text{nr}}+k_{\text{slope}}*{\text{CL}}_{\text{cr}}. \end{array}$$

In this analysis, the nonrenal elimination rate constant (*k*_nr_) was fixed to zero, assuming no nonrenal elimination of amikacin. Creatinine clearance was estimated from serum creatinine concentrations using the MM-USCPACK software [8]. 

The population analysis began with a uniform prior distribution spread over 40009 grid points of equal probability between stated initial ranges for each parameter. It then proceeded iteratively to maximize the likelihood of the entire model parameter distributions given the observed serum amikacin concentrations. The program stopped when 2 successive estimations of the log likelihood differed by less than 0.001%. This was the criterion that the likelihood function had reached a maximum. The program also estimated mean, median, and standard deviations of each pharmacokinetic parameter, as well as the covariance matrix of the model parameters. 

In addition, the nonparametric Bayesian posterior joint density was computed for each individual patient. In the nonparametric approach, this is done by computing the Bayesian posterior probability of each population model support point given the individual patient data. In this way, each patient's Bayesian posterior joint probability density is determined. 

### 2.8. Evaluation of the Parameter Estimates

The performance of the parameter estimates was evaluated by comparing the estimated serum amikacin concentrations with the patient's measured data. The bias (weighted mean error, ME) and precision (bias-corrected weighted mean squared error, MSE) were assessed according to Sheiner and Beal [13]. The bias and precision were evaluated separately for peak and trough serum amikacin concentrations.

## 3. Results

A total of 331 serum amikacin samples comprising 85 peak levels and 246 trough levels were analyzed (Table 1). The mean peak concentration was 22.32 *μ*g/mL (95% CI; 19.7–24.9 *μ*g/mL) and the mean trough concentration was 4.26 *μ*g/mL (95% CI; 3.88–4.64 *μ*g/mL). With the conventional amikacin dosage regimen, it was found that only 14% of the peak levels were within the therapeutic range of 30 to 50 *μ*g/mL, and only 24% of the trough levels were within the therapeutic range of 5 to 10 *μ*g/mL. This strongly suggests the advantage of population modeling and dosage individualization using tools such as NPAG and the MM-USCPACK software to maximize the precision of achievement of target serum amikacin concentrations.

### 3.1. Assay Error

The polynomial equation describing amikacin assay standard deviation (SD) was found to be

(3) $$\begin{array}{c} \text{SD}=0.2451+0.0950*C. \end{array}$$

The *C* ^2^ term was set to zero.

The coefficients of this equation were then entered into the MM-USCPACK software and used for weighting the data as described earlier, using the NPAG software.

### 3.2. Population Model Results

**Figure 1:**
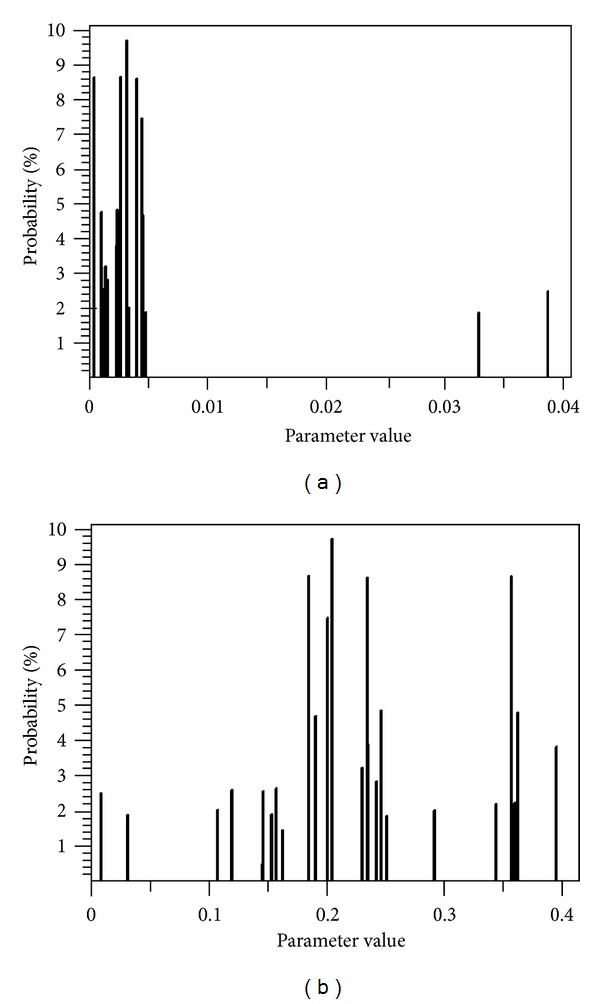
Marginal density plot of the parameters *k*_*s*_ (h^−1^ per unit of creatinine clearance); renal component of elimination rate constant (a) and *V*_*c*_ (L/kg); apparent volume of distribution of the central compartment (b) generated by NPAG program for adult ICU patients (*n* = 56) who received amikacin.

Using the NPAG software, convergence was reached on cycle 669. The final log likelihood was −777.4437, and the number of active grid points decreased from an initial value of 40009 down to 27. The parameter values were essentially stable before the convergence criterion was reached. The marginal density plots of the pharmacokinetic parameters *k*_slope_ and *V*_*c*_ are displayed in Figures 1(a) and 1(b). The pharmacokinetic parameter summaries estimated from the final population model are presented in Table 2. As shown in Figures 1(a) and 1(b), there may be two possible subpopulations of adult and elderly patients or subpopulations of patients with renally impaired and normal function. As shown in Figure 1(a), for instance, two subpopulations appear to be present, a principal one with *k*_*s*_ values ranging from almost zero to 0.005 h^−1^ per unit of creatinine clearance and another small one with values ranging from about 0.033 to 0.039 h^−1^ per unit of creatinine clearance. Moreover, it is possible that two subpopulations may also be present in the volume of distribution of the central compartment (*V*_*c*_). In this regard, the first subpopulation is centered at about 0.2 L/kg and another one centered at about 0.36 L/kg, a value seen somewhat more often in Caucasian ICU patients (Figure 1(b)).

**Figure 2:**
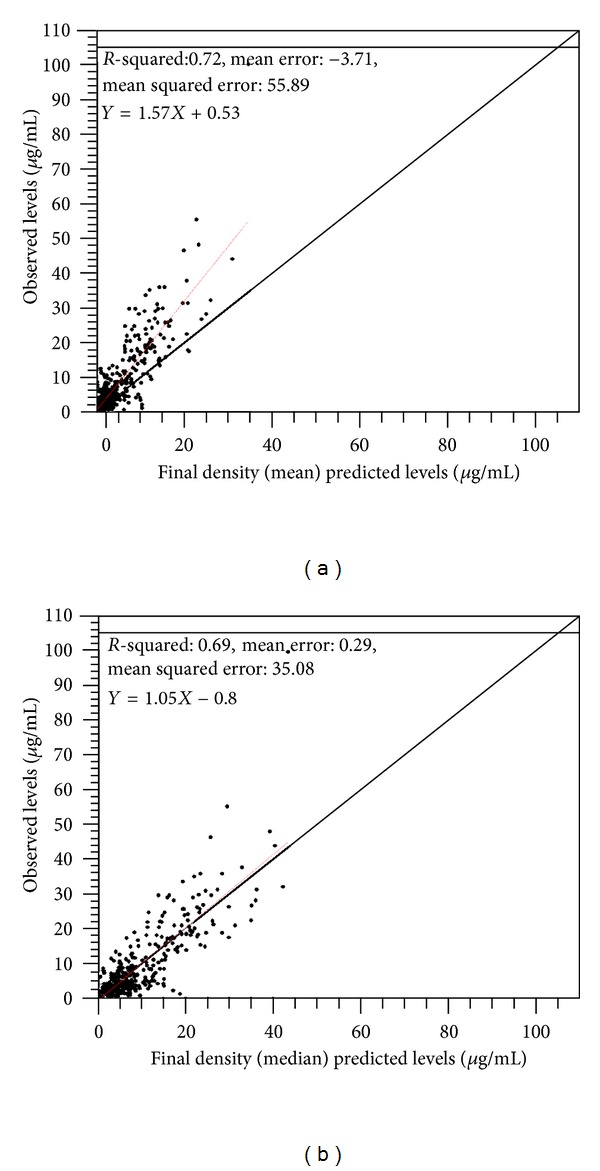
Scatter plot of predicted (*x*-axis) versus measured (*y*-axis) serum amikacin concentrations (*μ*g/mL) based on means (a) and medians (b) of population parameter distributions. Pooled data from all patients (*n* = 56).

**Figure 3:**
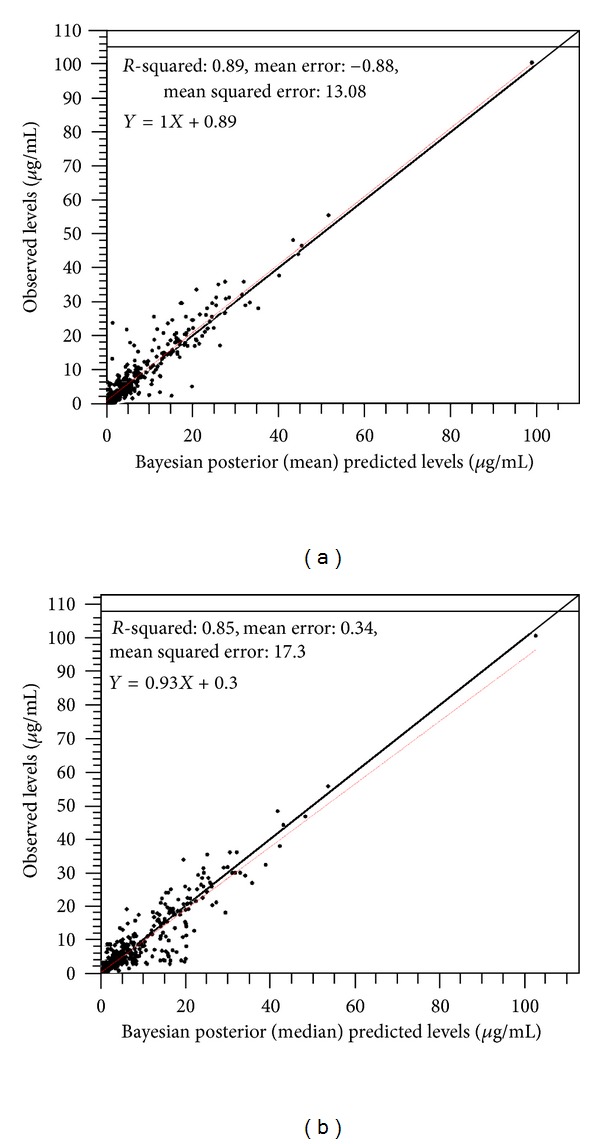
Scatter plot of predicted (*x*-axis) versus measured (*y*-axis) serum amikacin concentrations (*μ*g/mL) based on means (a) and medians (b) of each individual patient's Bayesian posterior parameter distributions. Pooled data from all patients (*n* = 56).

The performance of the NPAG population and individualized Bayesian estimates are shown in the plots of the observed versus estimated serum amikacin concentrations (Figures 2 and 3). The scatter plots of estimated versus observed serum amikacin concentrations using the mean (Figure 2(a)) and median (Figure 2(b)) population model parameter values have been demonstrated. The relationship between the mean predicted and measured serum amikacin concentrations was found to be the following: measured conc = 1.57  ∗  predicted conc + 0.53; *r* ^2^ = 0.72; ME = −3.71; MSE = 55.89 (Figure 2(a)), whereas the relationship between the median predicted and measured serum amikacin concentrations was found to be the following: measured conc = 1.05  ∗  predicted conc − 0.8; *r* ^2^ = 0.69; ME = 0.29, MSE = 35.08 (Figure 2(b)). Similarly, the scatter plots based on the means (Figure 3(a)) and medians (Figure 3(b)) of each individual patient's Bayesian posterior parameter distributions have been presented. In this regard, the relationship between the mean predicted and measured serum amikacin concentrations was found to be the following: measured conc = 1.00  ∗  predicted conc + 0.89; *r* ^2^ = 0.89; ME = −0.88; MSE = 13.08 (Figure 3(a)), whereas the relationship between the median predicted and measured serum amikacin concentrations was found to be the following: measured conc = 0.93  ∗  predicted conc + 0.3; *r* ^2^ = 0.85; ME = 0.34; MSE = 17.30 (Figure 3(b)). The estimates based on the individual patient's Bayesian posterior parameter distributions were much better than those based on the population distributions. This illustrates the utility of Bayesian individualization of each patient's model based on his/her individual data using TDM.

Separating peaks from troughs, the mean (±SD) observed and predicted (based on individual patient's mean Bayesian posterior parameter values) serum amikacin trough concentrations were 4.38 ± 3.26 *μ*g/mL and 4.14 ± 3.16 *μ*g/mL, respectively; whereas those for the peak concentrations were 23.36 ± 12.32 *μ*g/mL and 21.27 ± 12.42 *μ*g/mL, respectively. The mean error (ME) was lowest for amikacin trough levels and highest for peak levels (based on individual patient's mean Bayesian posterior parameter values), just as the assay error was less for the troughs and more for the peaks as described by the assay SD polynomial. The precision, mean squared error (MSE), of the mean serum amikacin concentration predictions (based on individual patient's mean Bayesian posterior parameter values) ranged from 4.67 *μ*g/mL (95% CI; 2.93–6.42) for trough levels to 22.16 *μ*g/mL (95% CI; 13.46–30.87) for peak levels, Table 3.

**Figure 4:**
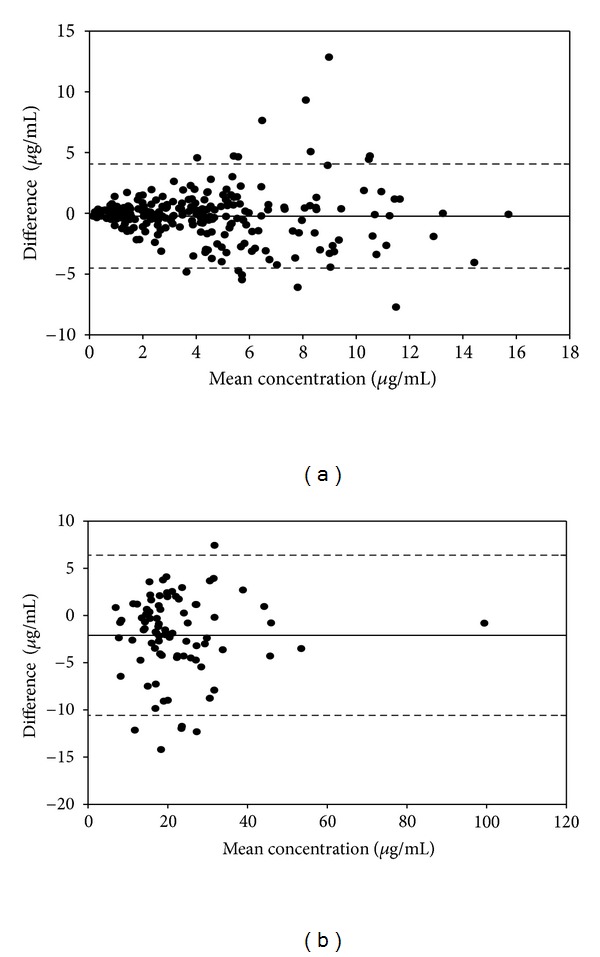
Bland-Altman plot of the mean serum amikacin (*x*-axis) trough concentrations (a) and peak concentrations (b) versus difference (*y*-axis) between predicted and observed amikacin concentrations. The solid line represents the mean difference; the dashed lines represent the limits of agreement (mean difference ±2 SD difference).

Bland-Altman plots of the mean serum amikacin trough and peak concentrations (based on individual patient's mean Bayesian posterior parameter values) are displayed in Figures 4(a) and 4(b), respectively, and are consistent with the relationship between the more precisely measured troughs and the less precisely measured peaks. As shown, 94% of trough residual levels and 93% of peak residual levels were in the range of the mean (±2SD) overall difference between estimated and measured serum concentrations.

**Figure 5:**
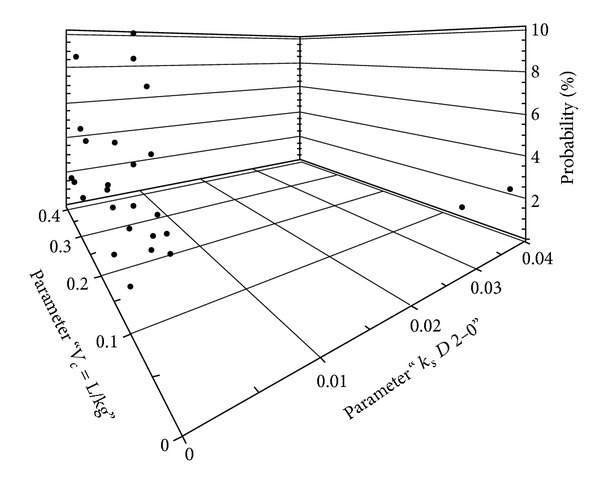
Three-dimensional plot of apparent volume of distribution of the central compartment (*V*_*c*_; L/kg) versus renal component of elimination rate constant (*k*_*s*_; h^−1^ per unit of creatinine clearance) for Kuwaiti ICU patients (*n* = 56) using NPAG program.

A three-dimensional plot of *V*_*c*_ versus *k*_*s*_ using NPAG software demonstrated the potential presence of two clusters of subpopulations (Figure 5). Moreover, a correlation exists between *V*_*c*_ and *k*_*s*_ (*r* ^2^ = 0.44).

## 4. Discussion

Amikacin pharmacokinetic parameter distributions have substantial interindividual variation among the patients treated with the drug. This interindividual variability is especially great in ICU patients [14], presumably owing to various physiological changes in ICU patients. The findings of the present study demonstrate that the *V*_*c*_ is not significantly increased in ICU patients, as its mean value of 0.23 L/kg (Table 2) is similar to that of normal patients and in agreement with previous studies [15]. However, except for our subgroup with the higher *V*_*c*_, our results contrast with those of other investigators who reported increased values of *V*_*d*_ in ICU patients [16–18]. Nevertheless, the narrow therapeutic range and great interindividual variability of amikacin in all patients emphasize the need for therapeutic drug monitoring of its peak and trough levels for optimized dosing as well as optimal efficacy and prevention of serious side effects. 

In this analysis, however, several possible subpopulations of patients were found, though there was no other information to identify why this should be so. The ability to detect such unsuspected subpopulations is a distinct strength of the nonparametric approach and is one of the reasons why this method of analysis was selected for use here. These subpopulations might well have been missed by population analyses using parametric approaches, which only compute means and covariances of the assumed distributions of the model parameter values [9]. The Bayesian posterior parameter values would have had to be analyzed, and the population analysis would then have to be specifically set up to detect such anticipated multimodal distributions. In addition, the problem of subpopulations within a larger population is most important from the point of view of developing maximally precise dosage regimens for patients. When a patient belongs to a larger population in which the parameter distributions are not Gaussian, as found here, the use of parametric models based on only single-point parameter estimates has no way to evaluate and optimize the expected precision with which a dosage regimen will hit a target. This is a distinct limitation of what is known as separation principle control [19]. This principle states that whenever one seeks to control a system, first by getting single-point estimates of the model parameter values (rather than estimating the entire parameter distributions) and then using these single point estimates to control the system, the control is done suboptimally. This is because there is no method of estimating the degree of failure of the regimen to hit the target. It is simply assumed that the regimen is designed to hit the target exactly, and everyone of course knows that this will not be the case. In contrast, nonparametric population models, having multiple discrete support points which describe the entire model parameter distributions without having to make any assumptions about their shape, can easily compute the expected weighted squared error with which any dosage regimen fails to hit a desired target and can then find the regimen which specifically minimizes that error. This is Multiple Model (MM) dosage design [9, 12] and it is well known in the aerospace community for flight control and spacecraft guidance applications. 

In the present RightDose clinical software, MM dosage design proceeds as follows: just as the process of weighted nonlinear least squares begins with an initial set of parameter estimates, so does MM dosage design begin with an initial estimate of the dosage regimen to be developed. This candidate regimen is given to each population model support point. Each point, having its own set of model parameter values, predicts future serum concentrations resulting from that regimen, with the probability estimated for each point in either the original population model or the Bayesian posterior joint density of an individual patient. At the time the desired target is to be achieved, one can compare each prediction, weighted by its own probability with the target goal, and the weighted squared error of the failure of that candidate regimen to hit the target is calculated. Then, just as in least squares, the dosage regimen is iteratively optimized until the regimen hitting the target with the minimum expected weighted squared error is found. This is MM dosage design. 

The combination of nonparametric population modeling and MM dosage design lends great strength to these applications. It can be seen that in addition to covariates, the MM dose regimen itself becomes a most important tool to minimize the variability of patient response when hitting a desired target goal. MM dosage design is unique to nonparametric models having their multiple support points. Such maximal precision can never be computed, and this process can never be done if only parametric models are used. In general, amikacin pharmacokinetic behavior in Kuwaiti ICU patients compares reasonably well with previously reported findings [10].  Table 4 presents the comparative mean population amikacin pharmacokinetic parameters of Kuwaiti patients in contrast to other ethnic groups including Hispanic, Asian, and Caucasian subjects. The results suggest that the Kuwaiti population is a part of a larger population comprising Hispanic, Asian, and Caucasian subjects.

In conclusion, a population pharmacokinetic model for amikacin was developed from 56 adult Kuwaiti ICU patients using the NPAG program. The present study demonstrates lack of significant differences in amikacin pharmacokinetic behavior in Kuwaiti patients in comparison with Asian, Hispanic, or Caucasian patients. The present study suggests that amikacin pharmacokinetics in adult Kuwaiti ICU patients are generally rather similar to those found in other patients. The distributions of the present population pharmacokinetic parameters can be utilized as population priors for developing initial amikacin dosage regimens in Kuwaiti patients, using the MM-USCPACK Rightdose PC software (http://www.lapk.org/). The findings of the present study will help clinicians to establish optimized amikacin dosing regimens for each individual Kuwaiti ICU patient, using MM Bayesian adaptive control. Further study is warranted to evaluate the clinical utility of these findings in Kuwaiti ICU patients.

## Figures and Tables

**Table 1:** Demographic and biological characteristics of the patients included in the population model.

Parameter	Mean (range)
Gender (male : female)	32 : 24
Age (years)	57.4 (19–90)
Weight (kg)	72.6 (50–110)
Height (cm)	166.2 (148–182)
Serum creatinine (mg/dL)	1.50 (0.60–6.26)
Creatinine clearance (mL/min)	75.06 (7.81–177.10)
Total no. of peak concentrations collected	85
Total no. of trough concentrations collected	246

**Table 2:** Population pharmacokinetic parameters of amikacin in 56 ICU patients.

Parameter	Mean	Median	S.D.	C.V., %
*k*_*s*_ (h^−1^ per unit of CLcr)	0.004045	0.002576	0.007054	174.389
*k*_12_ (h^−1^)	2.21207	0.584539	5.5061	248.912
*k*_21_ (h^−1^)	1.43121	0.23237	2.7957	195.338
*V*_*c*_ (L/kg)	0.23012	0.218929	0.08658	37.6236

**Table 3:** Assessment of absolute predictive performance of NPAG in amikacin population model. Figures in parenthesis are 95% confidence intervals.

Level	*N *	ME	MSE
T	246	0.24 (−0.03–0.51)	4.67 (2.93–6.42)
P	85	2.10 (1.18–3.01)	22.16 (13.46–30.87)

*N*: sample size; ME: weighted mean error; MSE: bias-corrected weighted mean squared error; T: trough; P: peak.

**Table 4:** Comparative mean population amikacin pharmacokinetic parameters for Kuwaitis (Arabs) and other ethnic groups.

Parameter	Hispanic*	Asian*	Caucasians*	Arabs**
*k*_*s*_ (h^−1^ per unit of CLcr)	0.00404	0.00424	0.00394	0.00405
*V*_*c*_ (L/kg)	0.248	0.264	0.26	0.23

*Jhee et al., 1994 [10].

**Present study.
